# A natural language processing approach for identifying temporal disease onset information from mental healthcare text

**DOI:** 10.1038/s41598-020-80457-0

**Published:** 2021-01-12

**Authors:** Natalia Viani, Riley Botelle, Jack Kerwin, Lucia Yin, Rashmi Patel, Robert Stewart, Sumithra Velupillai

**Affiliations:** 1 IoPPN, King’s College London, SE5 8AF London, UK; 2 South London and Maudsley NHS Foundation Trust, SE5 8AZ London, UK

**Keywords:** Psychiatric disorders, Schizophrenia, Data mining, Machine learning

## Abstract

Receiving timely and appropriate treatment is crucial for better health outcomes, and research on the contribution of specific variables is essential. In the mental health domain, an important research variable is the date of psychosis symptom onset, as longer delays in treatment are associated with worse intervention outcomes. The growing adoption of electronic health records (EHRs) within mental health services provides an invaluable opportunity to study this problem at scale retrospectively. However, disease onset information is often only available in open text fields, requiring natural language processing (NLP) techniques for automated analyses. Since this variable can be documented at different points during a patient’s care, NLP methods that model clinical and temporal associations are needed. We address the identification of psychosis onset by: 1) manually annotating a corpus of mental health EHRs with disease onset mentions, 2) modelling the underlying NLP problem as a paragraph classification approach, and 3) combining multiple onset paragraphs at the patient level to generate a ranked list of likely disease onset dates. For 22/31 test patients (71%) the correct onset date was found among the top-3 NLP predictions. The proposed approach was also applied at scale, allowing an onset date to be estimated for 2483 patients.

## Introduction

For mental health disorders such as schizophrenia, receiving timely and appropriate treatment is crucial. The duration of untreated psychosis (DUP) is defined as the period of time between the manifestation of first psychotic symptoms (*onset*) and initiation of treatment. Longer DUPs have been associated with poorer intervention outcomes, with worse recovery and global functioning^1,2^, as well as greater long-term social impairment^3^. There is a need for more comprehensive understanding of disease trajectories and prognostic monitoring on a population level. Research findings have had a considerable impact on mental health services in the United Kingdom, leading to the establishment of early intervention teams aimed at reducing DUP by supporting people with a first episode of psychosis (FEP). However, given the relatively low prevalence of schizophrenia, large-scale prospective clinical research studies are difficult to conduct.

With the growing adoption of electronic health records (EHRs), a wealth of patient-related information has become available for retrospective clinical research. In mental health EHRs, however, relevant disease onset information is mainly documented in the form of free text. To unlock clinically important information from EHR free text, natural language processing (NLP) approaches can be developed. These approaches have been successfully applied in a variety of clinical applications^4–7^, in particular for extracting specific types of clinical entities, e.g. symptoms, diagnoses and medications. Despite the increasing interest in, and promising future of, clinical NLP, also thanks to the open release of manually annotated corpora (e.g. intensive care unit notes, cancer reports)^8^ for the wider research community and NLP developers, most efforts have been directed towards specific types of clinical notes and use-cases. The transferability and generalizability of these approaches, however, is seldom evaluated in real clinical settings^9^, and there is still a need for the development of NLP solutions for more complex tasks such as temporal information extraction and relation recognition^7^.

In mental health EHRs, compared to other medical domains, clinically relevant information is predominantly documented in free text rather than in structured fields^10^. Unlocking this information for patient-level research can be particularly challenging, in that there is large variability in how concepts are defined and documented, potentially requiring different layers of information and relation extraction. To capture the information of interest, understanding the context of the documentation procedures and the underlying EHR data is essential when designing an appropriate NLP approach.

To enable the calculation of DUP at scale from EHR data, information on the date or age of psychosis onset needs to be extracted from free text. From an NLP perspective, identifying this construct on a patient level presents a number of challenges. First, psychosis onset mentions contain three core elements: a symptom mention (*hearing voices*), a temporal reference (*three years ago*), and a temporal association (*started hearing voices three years ago*). To extract each of these, a separate NLP approach could be potentially designed. Second, psychosis onset information is not systematically documented: it is usually reported only in specific types of EHR records during certain clinical assessment situations, but could also be repeated across multiple documents for a given patient, e.g. if the patient is seen by different services, the assessment is performed by different healthcare professionals, or in other healthcare pathway situations. Moreover, the information surrounding disease onset may be uncertain to the clinician (and, indeed, the patient) and there may be various types of relevant references related to onset (i.e. not only symptomology) and also conflicting statements in the records. Given this, the degree of uncertainty in determining disease onset is not just the ability for an NLP approach to discriminate correct information from available data, but there is also a degree of clinical uncertainty related to how information is reported and documented in clinical notes.

The extraction of clinical-temporal variables on a patient level requires analysing temporal associations within the texts. To model this problem, much NLP research has been directed towards *temporal information extraction*, addressing entity recognition and temporal link classification as separate, sequential, tasks^11–13^. First, symptoms and temporal expressions (i.e. strings representing a temporal reference, such as *in 2001*, or *three years ago*) are extracted as entities, then links between entity pairs are identified (e.g. BEGINS_ON). While this approach allows breaking down the problem into smaller steps, errors from the entity extraction phase might propagate and impact results on the subsequent detection of relevant temporal associations. Another approach such as the one proposed by Jensen et al. could be to identify relevant entities by mapping concepts with knowledge-bases (such as NOR-MeSH), and classify these for relevance in terms of current and past to generate patient trajectories^14^. This represents a non-trivial problem for concepts that are documented in many different ways, as in the case of positive psychosis symptoms. For example, the *delusion* symptom could be described with either specific keywords or longer phrases, especially when rephrasing past experiences reported by the patient (e.g. *He said he is constantly followed by a woman, who tries to steal his things. This woman has been with him since he was 19 years old*). Furthermore, there can be numerous clinically relevant entities in a clinical note, referring e.g. to the current clinical encounter, but entities relevant specifically to patient disease *onset* are likely only a small subset of all entities in a patient’s set of EHRs.

As an alternative NLP approach, the extraction of clinical-historical information could be modelled using larger units of text. Document-level classification approaches have been applied to different clinically relevant tasks, such as tumor status categorisation^15^ and medical sub-domain identification^16^, while sentence-level NLP models have been used for categorising medical conditions^17^. In the mental health domain, Jackson and colleagues used sentence extraction and classification to identify symptoms of severe mental illnesses starting from predefined keywords, thus targeting precision rather than recall (sentences without any relevant keyword were not included)^18^. Despite these recent advances in clinical text classification, most developed systems do not explicitly deal with temporal aspects and longitudinal narratives.

In previous work, we modelled symptom-time relations on an entity level (symptom keywords and temporal expressions)^19^, based on related work on temporal information extraction. While this approach was useful for understanding general symptom-time relations, and could be used for timeline reconstruction, it was likely to miss onset-related information that was expressed indirectly or that was mentioned in relation to a diagnostic term rather than a symptom. It also included symptom-time relations that were not related to disease onset, but instead references to e.g. the current clinical encounter. For example, symptom onset reported in the patient’s own words (*She said this presence has been around since the age of 16*) would not be captured by using standard keywords. Additionally, sometimes disease onset can be reported without reference to specific symptoms, e.g. for patients with a previously confirmed diagnosis from a different healthcare service.

In this study, we propose a novel solution to identify patient psychosis onset information from EHR text, using paragraphs and aggregating these to produce a ranked list of likely onset dates. First, we describe the process of creating and manually annotating a set of EHRs (*corpus*) for relevant disease onset information. Then, we present a hybrid approach for identifying paragraphs that are likely to contain psychosis onset information—combining a rule-based module for temporal expression extraction and a supervised machine learning model for paragraph classification. Finally, we propose a method to aggregate and rank all paragraphs extracted from each patient’s set of documents, thus producing a list of the most likely onset dates related to a patient. To the best of our knowledge, this is the first attempt at combining clinical and temporal NLP methods to generate patient-level disease onset information, and with that, to support advances in mental health research and care. The proposed approach was also applied at scale, allowing DUP to be estimated on a large patient cohort.

## Results

**Figure 1:**
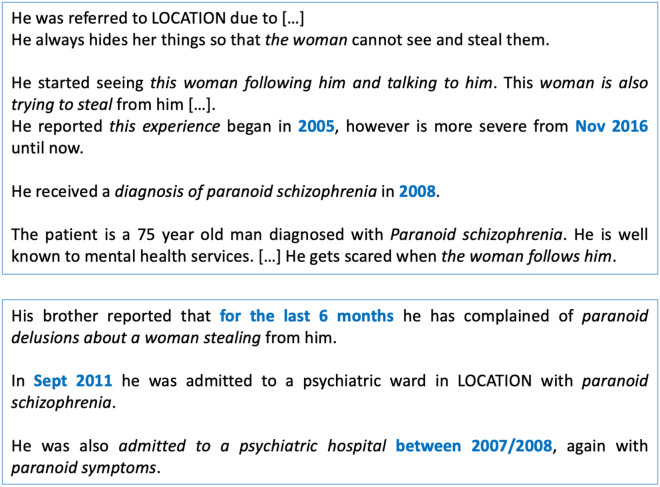
Example of a fictive patient’s EHR documentation including clear psychosis onset information as well as current symptomatology and past diagnoses. Potentially relevant clinical information is marked in italics, while temporal information is marked in blue.

We used data from the Clinical Record Interactive Search (CRIS) database^20^, which contains de-identified EHRs from the South London and Maudsley NHS Foundation Trust, a large mental health care provider in London. To build a corpus for NLP development, we extracted EHR documents for patients who had been diagnosed with schizophrenia, considering all patients referred after 1st January 2012 and looking at a 3-month window after the first referral date (see Methods for further details). Given the low prevalence of relevant disease onset mentions, retrieved documents were filtered with rule-based approaches (including document character length and presence of symptoms and temporal expressions), to only retain those that were more likely to include the information of interest. Figure 1 shows an example of potentially relevant information for one patient with schizophrenia, scattered across two different documents. In this case, documentation includes various references to psychosis-related information (marked in italics) including both past and current symptomatology, as well as past diagnoses. As shown here, psychosis onset is not always described with standard symptoms, but might need to be derived from reported diagnoses or non-specific behaviour.

Extracted documents were manually annotated for relevant onset information, thus developing a reference standard corpus. In this corpus, each document was then represented as a list of paragraphs with labels 1 (relevant to psychosis onset) or 0 (not relevant to psychosis onset).

To select candidate paragraphs for classification, we first applied a rule-based system for temporal expression extraction that we had previously adapted to the mental health domain (hereby referred to as SUTimeMentalHealth)^21^: this system identifies occurrences of temporal expressions within the text (*three years ago*, *at the age of 16*), and assigns a normalised value to them (*2017-08*, *AGE16Y*) (temporal expressions are marked in blue in Fig. 1). We only retained paragraphs that included at least one temporal expression, and then used these paragraphs to develop a supervised machine learning module for automated classification.

**Figure 2:**
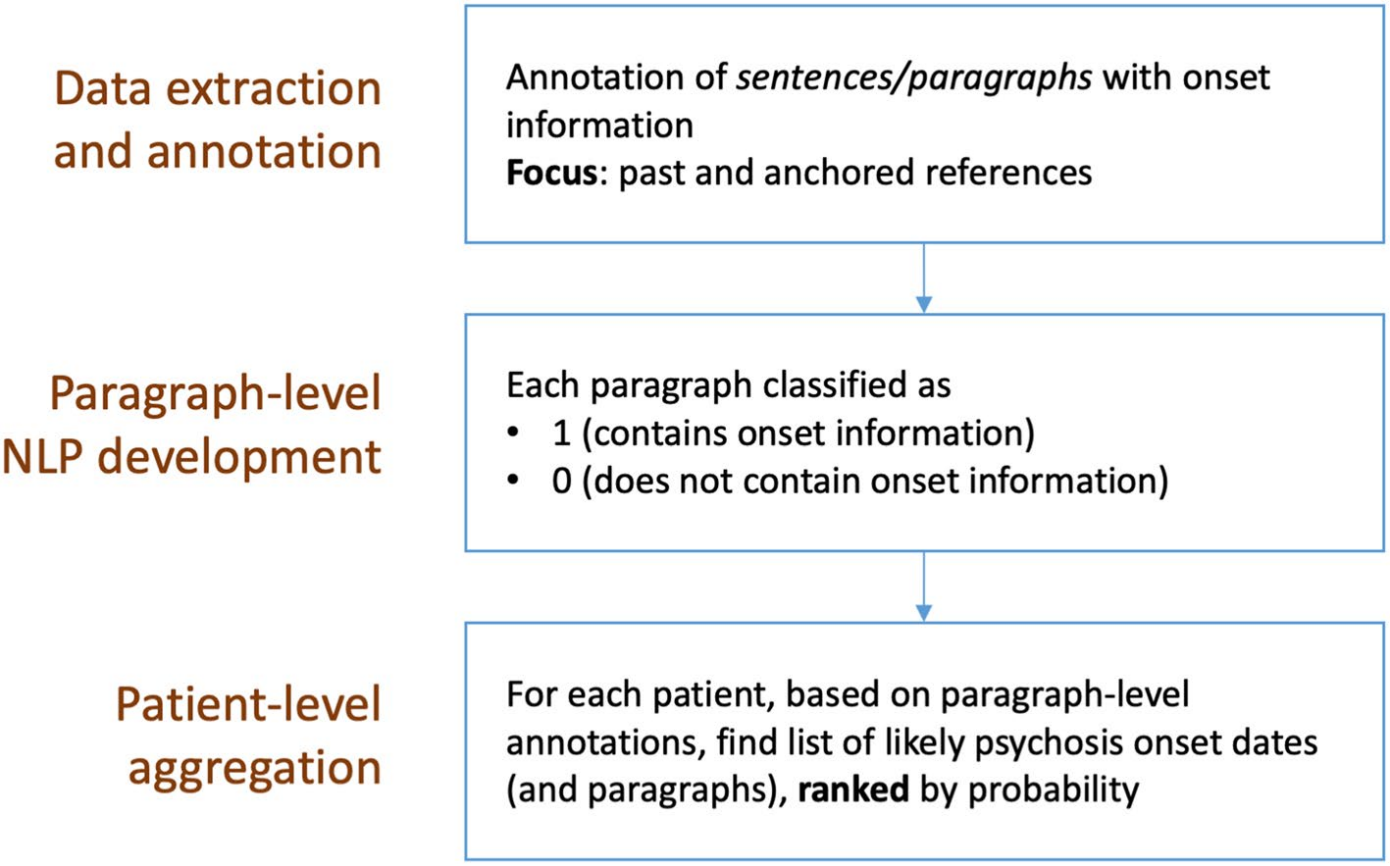
Main NLP development steps for determining onset information in mental health text: manual annotation, paragraph-level classification, and patient-level date ranking.

**Figure 3:**
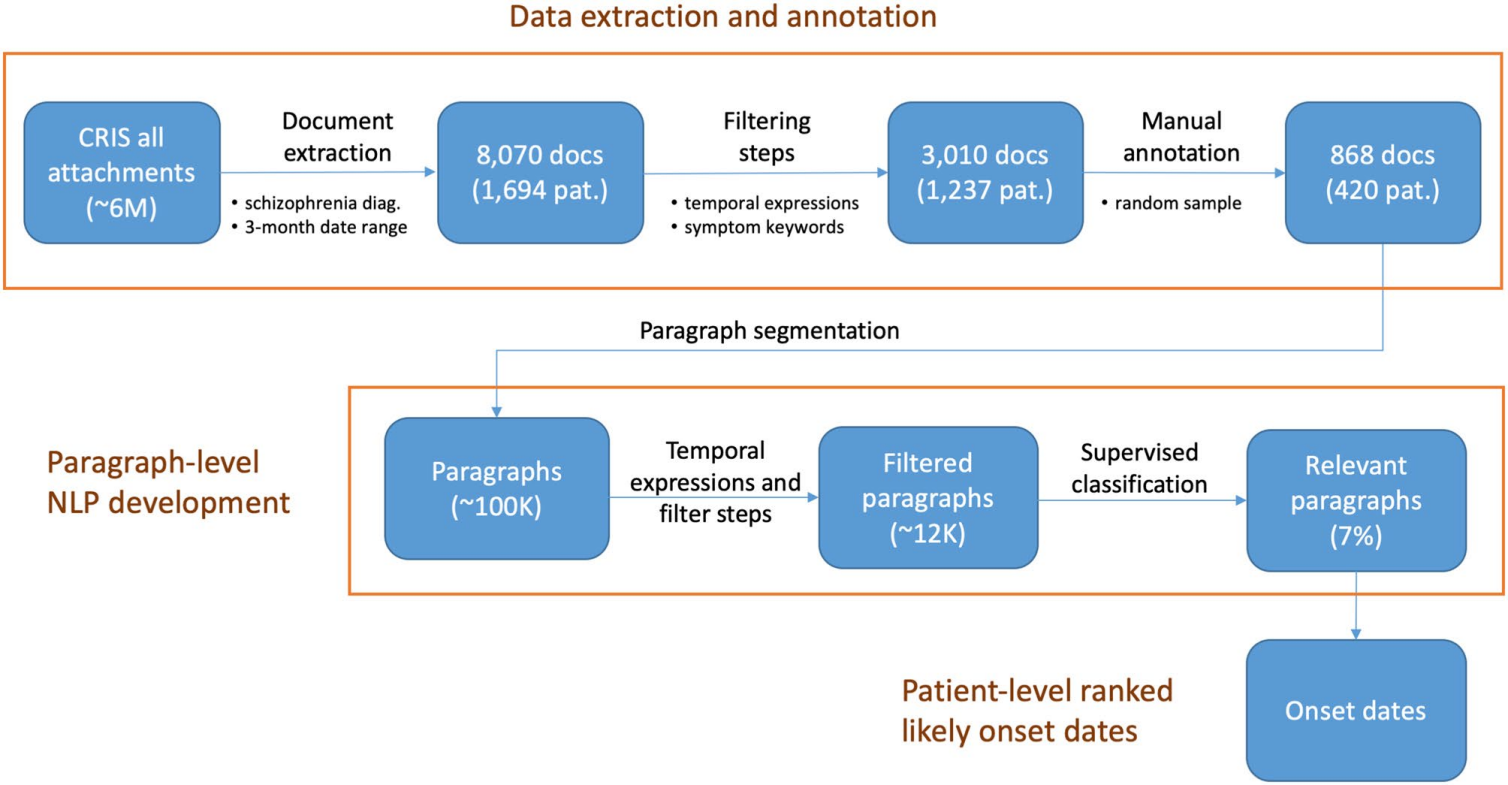
Data annotation and system development. Relevant documents were extracted and manually annotated for psychosis onset information. A paragraph classification model was developed on annotated data, with final aggregation on a patient level.

As a final step, we implemented an aggregation approach to combine paragraphs for each patient to produce a ranked list of likely disease onset dates. Figure 2 is a graphical representation of the main development steps. Figure 3 details some of these steps also in relation to the data we used for manual annotation and NLP development. The initial data extraction resulted in 8070 documents for 1694 patients. By applying document filtering steps, we obtained 3010 documents for 1237 patients.

### Annotated dataset for paragraph classification and patient-level aggregation

We randomly extracted documents following the filtering steps for 420 patients (approximately 34% of patients), this led to a total of 868 documents. A comprehensive annotation process was employed (further described in the Methods section). The primary task was to identify all relevant disease onset mentions, i.e. references to the emergence of disease, usually described within one or more sentences. Note that there could be more than one such reference/mention in each document. Each identified mention was also assigned a Time_information attribute (*past anchored*, *past not anchored* or *current*) and an optional normalised Value (e.g. *2012*, *AGE17Y*). To guide the identification of relevant onset mentions and understand the variability of these, annotators were also instructed to indicate a clinical Type (*symptom*, *diagnosis* or *non-specific*).

From the 420 patients and 868 annotated documents, we computed overall inter-annotator agreement (IAA) on a paragraph level and on a patient level. This resulted in a 0.55 F1 score for paragraph-level agreement, and a 0.85 time information agreement at the patient level (see Methods for further details). We also computed Time_information and Type attribute agreement on overlapping annotations, which resulted in a 0.92 score for the *pastanchored* value.

To create the final corpus for the paragraph classifier, we only retained *past anchored* annotations with a specified temporal Value. Given the low prevalence of onset mentions, we did not use the clinical Type sub-groups for this part, and addressed paragraph classification as a binary task (relevant or not relevant for psychosis onset).

The corpus with only *past anchored* annotations included a total of 1047 onset annotations (for 344 unique patients, 82%). We matched these to corresponding paragraphs, and filtered them based on paragraph length and temporal expression count. Specifically, we only kept paragraphs with at least one temporal expression, as extracted from SUTimeMentalHealth. This led to 12,168 paragraphs with a total of 806 positive labels (7%), which were split into training set (274 patients, 10,195 paragraphs) and test set (70 patients, 1973 paragraphs).

For patient level aggregation evaluation, we created an adjudicated reference standard on 50% of the annotated data (210 patients). The adjudication task was to resolve annotations with disagreement and assign one normalised temporal value at the patient level (onset date) per clinical type, if present, i.e. there could be three unique onset dates per patient in theory. 151 out of the 210 patients had at least one *past anchored* onset date (72%), with an average of 1.4 unique dates/ages per patient.

### Onset paragraph classifier

Classifier model selection was performed with a 5-fold cross validation on the training set, while final performance was evaluated on the test set.

In Table 1, we report the main results from paragraph classification experiments (averaged across 5 folds on the training set). The following machine learning classifiers were compared: logistic regression (LR), random forests (RF), and support vector machines (SVM). Two different text representations were compared: term frequency-inverse document frequency (TF-IDF) counts and word embeddings. Additional features (*count*) were also included based on paragraph length and presence of symptoms and temporal expressions. To deal with class imbalance, random subsampling was performed on the majority class.

**Table 1:** Paragraph classification results.

Features	Model	P	R	F1
Emb + count	LR	0.228	0.794	0.352
	RF	0.350	0.564	0.426
	SVM	0.202	0.836	0.326
Emb	LR	0.168	0.806	0.28
	RF	0.328	0.542	0.404
	SVM	0.166	0.84	0.278
TF-IDF + count	LR	0.290	0.724	0.412
	RF	0.324	0.694	0.438
	SVM	0.274	0.766	0.404
**TF-IDF**	**LR**	**0.272**	**0.786**	**0.404**
	RF	0.326	0.644	0.432
	SVM	0.270	0.758	0.398

*P* precision, *R* recall, *F1* F1-score for different classification algorithms: *LR* logistic regression, *RF* random forests, *SVM* support vector machines.

As mentioned, our end goal was to extract onset dates on a patient level. Since first referral documents typically include a high number of temporal expressions (65.6 on average^21^) and these expressions can relate to many various clinical events (not only disease or symptom *onset* information), identifying the most likely onset date is a challenging task—especially considering the presence of multiple documents for each patient (2.4 on average). To achieve this, an ideal paragraph classifier should not miss any relevant instances that might contain the correct onset date. For this reason, we used recall as the main performance metric for the paragraph classification task. At the same time, we wanted to keep a reasonable F1 score (≥ 0.4), given the intrinsic complexity of the NLP task. Overall, using TF-IDF features resulted in slightly higher F1 scores as compared to word embedding features. Moreover, adding count features did not seem to bring major improvements in terms of recall. Taking this into consideration, we selected the TF-IDF LR model for further patient-level experiments (highest recall among TF-IDF models).

To assess the effect of random subsampling, we also ran experiments by using all training data with balanced class weights. For the LR and SVM models, this resulted in an improved F1 score (0.450 and 0.448, respectively) but a drop in recall (0.668 and 0.530, respectively). For this reason, we decided to keep random subsampling as our preferred approach. Of note, recall values when using all training data and no class weights were 0.070 and 0.116, respectively.

### Patient-level aggregation and ranked onset dates

The supervised paragraph classifier generates multiple outputs for each patient (either within the same document or across multiple documents). To combine these outputs on a patient level, we developed an heuristic approach, ranking all temporal references according to the classifier probability. The predicted dates or ages were in the form of a ranked list (with an average of 3.2 predicted dates/ages per patient, range 0–15). For each patient, we selected the top N highest scoring predictions, and then computed a *Top-N match*: if any of these predictions matched at least one reference onset date (i.e. same year or age), we considered the patient-level extraction to be correct.

Table 2 presents results for the patient-level aggregation task, evaluated on top 1, 3, and 5 dates or ages produced by our heuristic approach (*Top-N match predictions*). Among the 151 patients with a *past anchored* onset, 120 belonged to the training set, and 31 to the test set. For training set results, we used the same cross-validation setup as for paragraph classification experiments: for each fold *i = 1..5*, we used the model trained on the remaining 4 folds to identify relevant paragraphs, i.e. prediction performance was calculated on the one fold not used for training the model. For test set results, we applied the paragraph classification model trained on the training set.

Given the high degree of clinical uncertainty around documented onset dates, we considered that an NLP approach that is able to propose three possible dates (top-3 evaluation) would be helpful to reduce the time required for manual review and enable research on a large scale. This resulted in a test score of 0.710, corresponding to a mean precision and recall @3 of 0.537 and 0.599 respectively. For comparison purposes, we also evaluated a baseline approach that returns the top-3 earliest dates from any of the predicted paragraphs, which resulted in a test score of 0.645.

To investigate how many mismatches were due to errors from the paragraph classifier (either false positives or false negatives), we also applied our aggregation approach to the reference paragraphs, i.e. using paragraphs labelled as 1 from manually annotated onset mentions (*Top-N match reference*). We calculate a relative match by dividing the number of matched patients from predictions and the number of matched patients from reference paragraphs (*Relative match*). Assuming the aggregation step can correctly extract and rank onset dates from a reference set of paragraphs, this value estimates the ability of the paragraph-level classifier to identify relevant spans of text.

To gain more insight into potential issues with our approach, we manually reviewed the 9 patients for which the onset date could not be found (in the test set). For 4 patients, no paragraph was found at all: missed references included relative temporal expressions (*3 year history of hallucinosis*) and vague symptom references (*this woman has been with him for 3 years*). For 4 patients, extracted paragraphs were relevant but did not correspond to disease onset (*in 2009 he was referred again to hospital, after the visit of the community mental health team*; *I have known this patient since Nov 2015, when she was first seen by this team*). For the last patient, the extracted paragraph was related to the current mental state rather than the first emergence of symptoms (*he reports his mental state has been stable for the past six months*).

**Table 2:** Patient-level aggregation results: from the highest scoring predictions, a match is counted if the predicted value matches a value in the adjudicated reference data (top-N match).

Set	Model	Top-N match predictions	Top-N match reference	Relative match
Training	Top-1	75/120 (0.625)	83/120 (0.692)	0.904
	Top-3	87/120 (0.725)	92/120 (0.767)	0.946
	Top-5	92/120 (0.767)	94/120 (0.783)	0.988
Test	Top-1	19/31 (0.613)	25/31 (0.806)	0.760
	Top-3	22/31 (0.710)	26/31 (0.839)	0.846
	Top-5	22/31 (0.710)	26/31 (0.839)	0.846

### Implications beyond the reference dataset

We first evaluated our proposed NLP approach on an independent set of patients and documents. We extracted and filtered available documents for 20 randomly selected patients who were not included in our reference dataset, and applied the developed paragraph classifier on these (only considering paragraphs with at least one temporal expression as extracted by SUTimeMentalHealth). Predicted paragraphs were manually reviewed by one psychiatrist and one medical student: 68 out of 92 (74%) paragraphs were marked as relevant, and 54 of these (79%) were marked as referring to the correct onset date. Non-relevant paragraphs were mostly related to past life events not necessarily related to schizophrenia (*background of type 2 diabetes*) or current symptomatology (*On assessment, she reported hearing whispering voices*).

Our proposed approach was then applied on a large patient cohort, selecting all patients who had received a diagnosis of schizophrenia, and had documentation in the period 01/01/2007–31/12/2019. Also in this case, we considered a 3-month window after the first referral date. This resulted in 4645 patients and 23,773 documents. After applying the rule-based document filtering (document length, presence of symptom keywords and time expressions), we retained 3357 patients (72%) and 7993 documents, which were divided into paragraphs. Again, we used SUTimeMentalHealth for temporal expression extraction, and the developed LR classifier for paragraph classification. By applying the patient-level aggregation approach and cleaning extracted dates (e.g. we removed those that were after the first admission date), we were able to estimate ranked onset dates for 2483 patients (53% of all initial patients).

Since multiple dates could be extracted for a single patient, we retained a maximum of 3 results each (note that these multiple dates were usually within 0–3 years from each other). As a final step, DUP was calculated as the difference between the first referral date and the estimated onset date.

As a means of validating our overall NLP approach, we computed mean estimated DUP in patients who had been accepted to early intervention services for psychosis (FEP teams) vs. other teams. Out of the 2483 patients with an estimated onset date, 578 (23%) were seen by at least one FEP team within 3 months of the first referral date. In this group, the estimated minimum DUP was 0.9 years. Conversely, the estimated minimum DUP for patients seen by other teams was 3.4 years. Table 3 reports mean DUP values in the two groups, considering either the minimum or the maximum estimated DUP for each patient.

**Table 3:** Comparison of estimated DUP characteristics for FEP teams vs. other teams.

	DUP min		DUP max	
	FEP teams	Other teams	FEP teams	Other teams
Patients	578	1835	578	1835
Mean DUP	0.9	4.2	3.4	8.2
Std DUP	3.8	8.4	6.3	11.3
Range*	0–52	0–61	0–52	0–72

*We considered DUP values of − 1 as 0-year durations (i.e. 1-year error margin).

## Discussion

This study presents an hybrid NLP approach for extracting the date of psychosis onset from mental health records, to enable clinical research using this variable on a large scale. Given the complexity of this NLP problem, where the relevant information could be written within multiple patient documents, with different levels of clinically relevant detail, and with different temporal references, we (1) defined an annotation schema to allow identifying various types of onset information, (2) employed a paragraph classification model that does not explicitly rely on clinical entities such as symptom keywords, (3) proposed an aggregation approach to retrieve a list of likely onset dates at the patient level. To the best of our knowledge, no other work has used NLP methods to retrieve complex clinical-temporal patient variables from mental health records. An overview of performed steps is available at: https://github.com/medesto/onset_pipeline.

To simplify the manual annotation task, we instructed annotators to identify the most relevant onset mention per type and per document. However, given the low prevalence of onset mentions across documents (2.2 per document), this was not trivial. IAA on the paragraph level was 0.55, indicating that many onset mentions were single-annotated; however, since most of these were then retained during the adjudication phase, we concluded missing annotations were due to uncertainty/missed information rather than non-relevant mentions. This could be expected considering the complexity of mental health EHRs, where there is variability in document types and the language used to describe symptoms. As a matter of fact, agreement on time information at the patient level was much higher (0.85), indicating that it is possible to aggregate sparse paragraph information for a given patient. Moreover, the breakdown of IAA per attribute revealed that the best agreement was obtained on *past anchored* (0.92) annotations. This is an encouraging result, as our ultimate goal is to provide a means of extracting definite (and past) psychosis onset dates.

The motivation for using paragraphs as the unit of analysis is multi-fold. First, compared to other clinical domains, mental health EHR documents are particularly complex: they rarely include predefined sections, and information on past and current symptomatology is often documented in different parts of the text . For this reason, employing a document-level classification approach would make it hard to extract specific clinical-temporal variables. At the same time, psychosis onset references typically span one or more sentences, as two different pieces of information need to be captured: the actual symptom or diagnosis, which can be described in detail (including the patient’s own words), and the temporal reference, which could be vague or related to a particular event in the patient’s life, and could be mentioned earlier in a paragraph. For these reasons, we considered paragraphs to be an appropriate unit of analysis, as they are likely to cover the information needed to determine disease onset.

To address the identification of paragraphs containing clinical and temporal information, we used an hybrid approach leveraging both rule-based information extraction (for temporal expressions) and supervised machine learning (for paragraph classification). The rule-based step was needed to extract specific dates and their normalised values from the text. The supervised classification model was useful to capture different ways of documenting disease onset.

To develop our paragraph classifier, we experimented with different features and machine learning models. From Table 1, we can observe that most models achieved a relatively high recall. However, precision values were low across all combinations. To investigate this, we manually reviewed some of the false positives from our selected classifier (LR algorithm with TF-IDF features). Most instances included relevant psychosis symptoms that, however, were not directly related to disease onset. An example of this is given by: *He was referred to LOCATION due to [...] He always hides her things so that the woman cannot see and steal them*. The paragraph describes ongoing delusional behaviour, but it also includes a reference to a past clinical event. In some cases, reviewed false positives included both a symptom mention and its temporal reference; however, this would not necessarily represent the *first* onset mention (if earliest symptoms were documented within the same text).

Identifying a single onset date for a given patient is not a trivial problem, even assuming all onset mentions are correctly extracted. Taking this into account, we modelled onset date extraction as a ranking problem, using some heuristics to combine extracted paragraphs and ranking them according to the classifier’s probability. Results in Table 2 are promising, especially looking at patient-level matches when using predicted vs. reference paragraphs (0.846 top-3 match on the test set). From our error analysis, future development will be needed both on paragraph classification and patient-level aggregation. In particular, this will involve: (1) modelling and extracting vague temporal references, (2) exploring other text representations to capture unusual symptom/non-specific mentions, and (3) investigating more refined approaches to extract onset dates from relevant paragraphs. As mentioned, determining disease onset from mental health records entails dealing with some degree of clinical uncertainty, related to how information is reported and documented. This means that even an information extraction approach which is 100% accurate may not yield completely accurate onset data. Given this, an automated approach to extract these data in a ranked, aggregated way may not only be helpful in itself but may also assist clinicians and researchers in understanding the degree of uncertainty surrounding disease onset and help to reduce this. As a proof of concept, we applied the developed approach on a cohort of 4645 schizophrenia patients, to investigate to what extent it could be used to retrieve DUP information. By applying the rule-based filtering steps and the subsequent NLP approach, we were able to estimate an onset date for 2483 patients. This is an encouraging result, considering that not all patients have in fact a documented past onset (from our manual review, about 14% of patients had a current or non-documented onset). For 75% of identified patients, the estimated dates were in the range of 0-3 years, which represents a fair error given the degree of uncertainty around this variable. The suggested validation on data from different teams (FEP teams vs. others) indicates that our NLP approach has the potential to correctly identify marked differences in DUP values. As a next step, further quantitative evaluation will be performed, to assign a level of confidence to predictions, and assess how these can be used to support advances in mental health research.

This study presents some limitations with respect to dataset creation and NLP development. First, despite the considerable effort put into manual annotation, the prevalence of onset mentions is low in our dataset. This could be due to the EHR documentation itself, where psychosis onset dates are not always documented in a straightforward way, or to the filtering steps that were applied during data extraction. Second, we further filtered documents by using SUTimeMentalHealth, which might have missed relevant information. However, these filters were needed to make the manual annotation task feasible and allow extracting actual onset dates (with normalised values). From the NLP point of view, we used relatively standard classifiers, without relying on complex linguistic features. Despite this, we obtained promising results; combining extracted paragraphs on a patient level, we were able to correctly identify onset dates in 71% of patients.

## Methods

### Dataset extraction and filtering

The CRIS database is derived from the electronic Patient Journey System (ePJS), a comprehensive electronic healthcare record system where all patient-related information is recorded, including structured fields (e.g. medication, diagnosis) and unstructured data. Within ePJS, different types of free text documents are included, ranging from longer attachments (e.g. referral letters, clinical assessments) to shorter event comments (e.g. daily notes). To create a corpus for NLP development, we focused on patients with a diagnosis of schizophrenia (*diagnosis like %f20% or %schizophrenia%*) who were referred after 1st January 2012. We extracted all attachments written within three months of the first referral date, as this period would most likely contain documentation about patients’ history. We excluded patients who had documentation from early intervention services for psychosis as these patients are admitted to these services following a first episode of psychosis, i.e. disease onset is close to admission date.

Following our previous work, attachment documents were filtered by length (character length > 50% percentile), presence of symptom keywords (at least one), and temporal expressions (more than five expressions)^21^. Symptom keywords were selected with the help of two psychiatrists, using an initial list of 56 symptoms (list A) and an extended list of 133 keywords derived from word embedding techniques (list B)^22^. Temporal expressions were extracted using SUTimeMentalHealth^21^. From the filtered dataset, we randomly extracted 420 patients for manual annotation, for a total of 868 documents (Table 4) with an average length of  2300 words.

**Table 4:** Dataset extraction and filter steps.

Dataset	Documents	Patients
Initial extraction	8070	1694
Filtered data (list A)	2662	1188
Filtered data (list B)	3010	1237
Corpus for annotation	868	420

### Manual annotation

For each document in the corpus (868 documents in total, 420 patients), annotators were instructed to identify the most relevant onset mention per clinical type. Each mention was assigned the following attributes (Table 5): Time information (temporal reference of the onset mention), Value (string describing the actual date or age, if any), and Type (clinical type related to the identified mention).

All documents were double annotated by medical students (in their 3rd/4th year). To guide the annotation task, we also developed specific annotation guidelines, including examples and symptom keywords to be used as a reference. Annotation guidelines are publicly available at https://github.com/medesto/onset_pipeline.

Adjudication was conducted on documents from 210 patients: the adjudicator reviewed all available annotations, selecting which ones to keep and updating attribute values if needed. In addition, one specific date or age per onset type was selected, both on a document level and on a patient level (picking the earliest dates across documents). The finalised onset dates were used as the gold standard for evaluating the aggregated, ranked paragraphs (with an average of 1.4 dates per patient).

**Table 5:** Annotation schema for identifying mentions of psychosis onset.

Attribute	Possible values	Description	Example
Time information	Past anchored	Referring to a definite past time	She started hearing voices at the age of 8
	Past not anchored	Referring to an indefinite past	History of having delusional beliefs
	Current	Related to the current presentation	Patient presenting with auditory hallucinations. This is clearly a first psychosis episode.
Value	YYYY-MM-DD; AGE-number-Y	String describing the actual date or age for past anchored mentions	2012; AGE8Y
Type	Symptom	Clearly defined positive psychosis symptoms	Hallucinations
	Diagnosis	Diagnoses related to schizophrenia	Paranoid Schizophrenia
	Non-specific	Vague clinical concepts indicative of psychosis	Strange behaviour

92% of these documents (800) had at least one onset annotation, corresponding to 95% of the patients (400). On average, there were 2.2 onset annotations per document (1768 in total).

**Figure 4:**
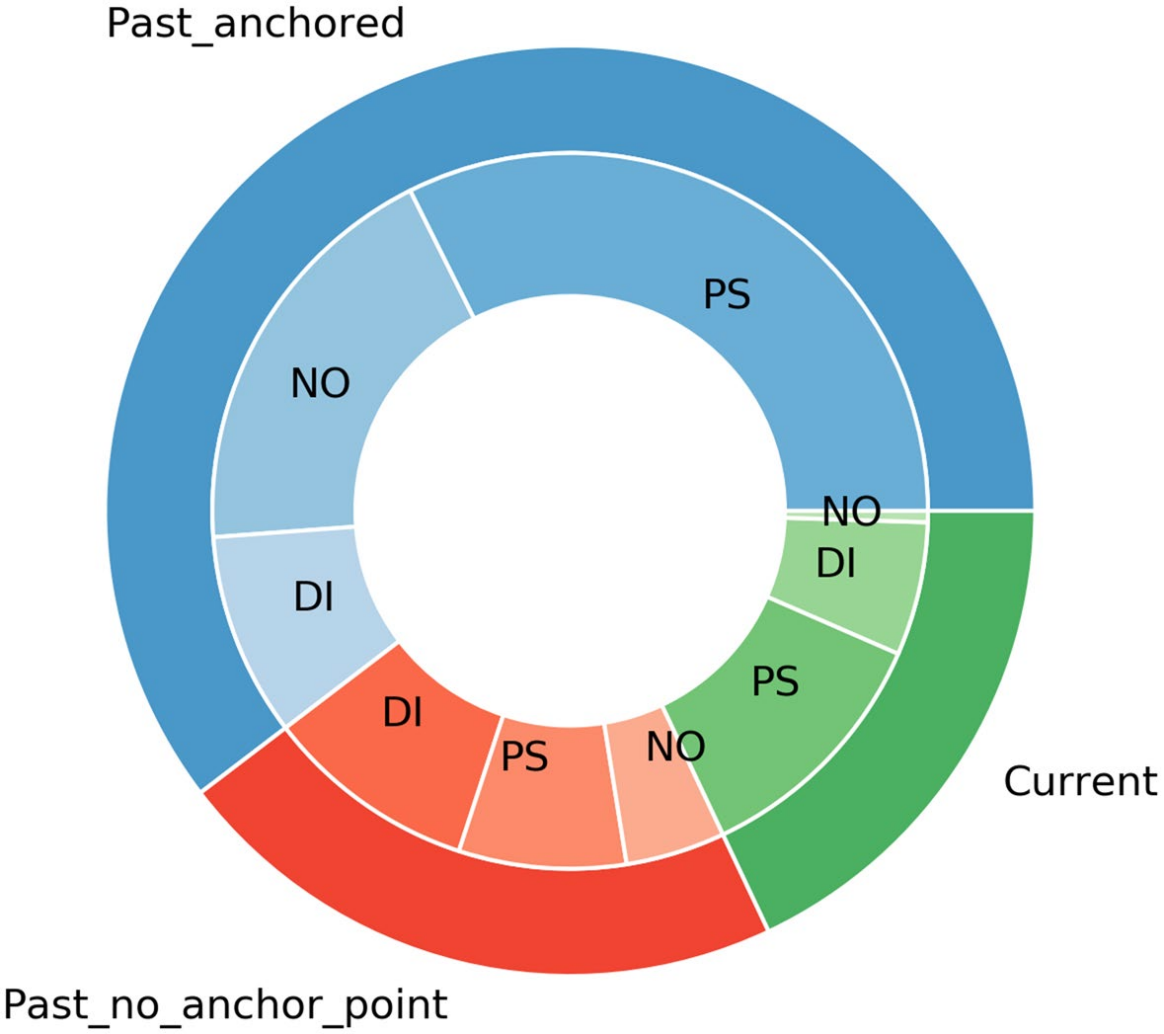
Distribution of the Type attribute in relation to Time_information (adjudicated dataset). *PS* psychosis symptom; *DI* diagnosis; *NO* non-specific. Past_anchored annotations (60%) were mostly related to symptoms (54%) and non-specific instances (31%). Past_not_anchored annotations (22%) had a higher number of diagnoses (44%).

IAA for onset mentions was measured at the paragraph level. For the Time_information and Type attributes, we calculated agreement on overlapping annotations, i.e. those that were marked by both annotators (670). Adjudication was conducted on documents from 210 patients (50%). Table 6 reports frequency and IAA for each annotated item (attribute frequency was computed on the adjudicated dataset). Figure 4 shows the distribution of the Type attribute in relation to Time_information.

**Table 6:** Dataset annotation.

Item	Counts	IAA
Onset mention spans	1768	0.55 (0.92*)
(Overlapping)	(670)	
Past_anchored	60%	0.92
Past_not_anchored	22%	0.66
Current	18%	0.68
Symptom	51%	0.86
Diagnosis	25%	0.78
Non-specific	24%	0.62

Number of onset mentions in the whole annotated dataset (total number and overlapping set), and attribute frequency in the adjudicated dataset (Time_information and Type).

*IAA* inter-annotator agreement.

*Indicates agreement between the adjudicator and the union of annotations.

Computing IAA on a paragraph level resulted in a 0.55 F1 score. Since we were interested in determining a patient-level variable rather than single onset mentions, we did not consider this to be a problem. While some mentions could be easily missed (especially considering the low prevalence of onset paragraphs), the underlying reference could be repeated in different parts of the document (or even different documents) and be therefore captured by our approach. Hence, we used the union of all annotations for NLP development. Of note, when computing the agreement between adjudicated mentions and the union of annotations, we obtained an F1 score of 0.92.

Given the nature of our problem, we also computed patient-level agreement using a set of heuristics based on the Time_information attribute:

- if at least one *past anchored* mention is found, the patient-level classification is *past anchored*
- else, if at least one *past not anchored* annotation is found, the overall classification is *past not anchored*
- if all annotations are *current*, the overall classification is *current*

Under these assumptions, the final agreement was 0.85.

As regards patient-level adjudication, 151 out of 210 patients had a *past anchored* documented onset (72%); 31 were classified as *past not anchored* (15%) and 14 as *current* (7%). For the remaining 14 patients (7%) no onset information was available at all.

**Figure 5:**
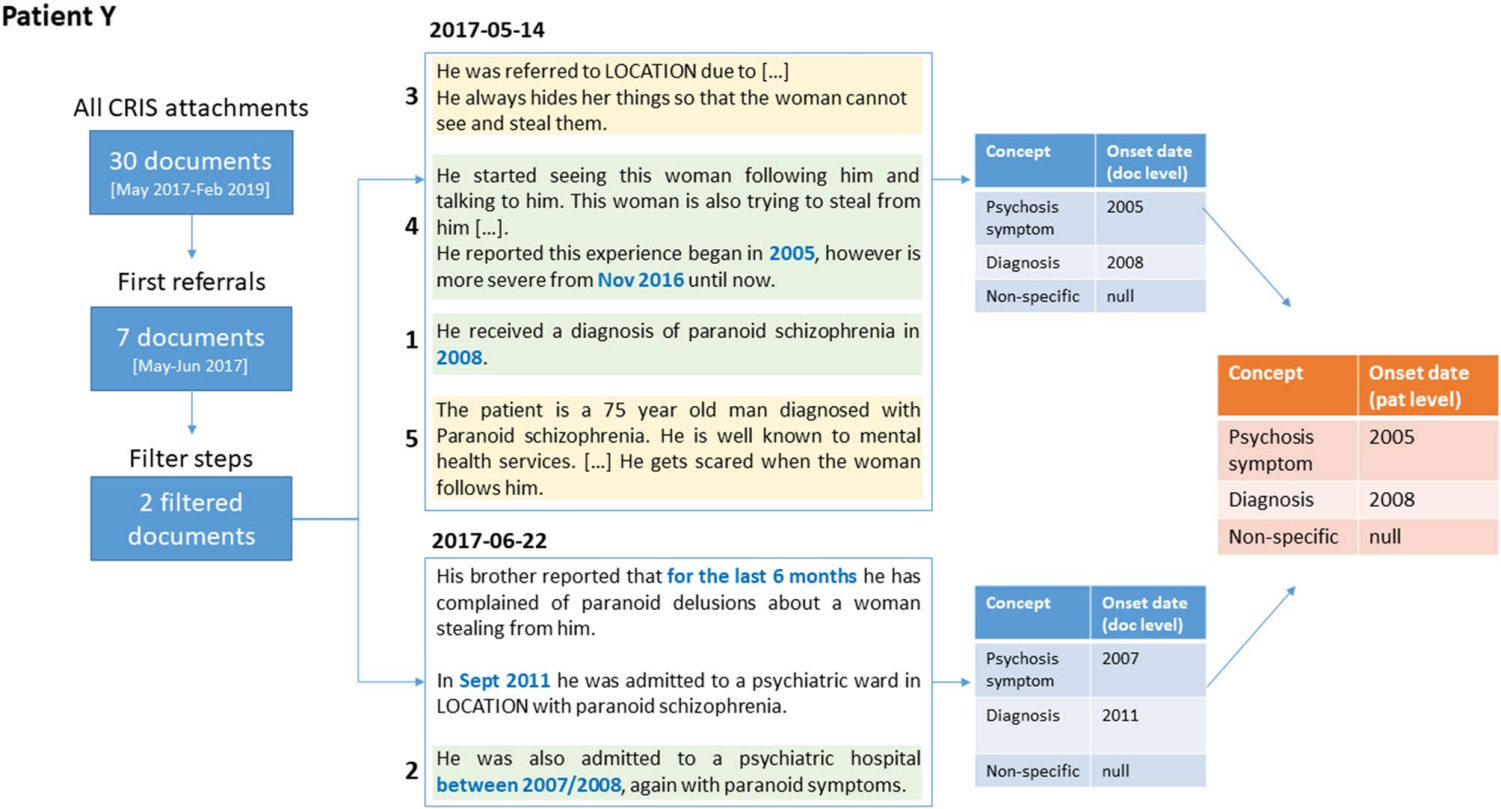
Data extraction and annotation for one fictitious patient. First referral documents are extracted from CRIS and filtered according to length, temporal expression count and symptom count (left). Documents are then annotated for relevant onset mentions, with types ‘psychosis symptom’, ‘diagnosis’ or ‘non-specific’ (center). In the adjudication phase, an onset date is selected per each type, first at the document level and then at the patient level (right). Paragraphs identified by the NLP classifier are marked in green (true positives) or yellow (false positives): the reported number represents their ranking.

Figure 5 shows the document extraction, annotation steps, and paragraph classifier results for one fictitious patient. Starting from all available CRIS attachments (30 documents from May 2017 to February 2019), first referral documents are selected (7 documents in the period May-–June 2017) and filtered according to length, temporal expression count and symptom count. The two filtered documents are then annotated for relevant onset mentions. Each document is represented as a list of paragraphs: the relevant ones are those including also temporal expression annotations (which are highlighted in blue and bold). In the adjudication phase, onset dates are chosen both at the document and the patient level (right side of the figure). Note that this example represents a challenging case, where a different value might be chosen depending on the particular document.

### NLP development

For NLP development, documents were divided into paragraphs using HTML tags, and each paragraph was labelled as 1, if containing a *past anchored* annotation, or 0 otherwise. At this stage, we did not distinguish between different onset types. Despite the utility of these for guiding the manual annotation process, the low prevalence for each of them made it unfeasible to develop type-specific classifiers. Moreover, distinguishing between clinical types is not always straightforward, especially as regards positive psychosis symptoms vs. non-specific mentions^22^. To mitigate the class imbalance problem, we filtered examples by removing short and headers-like paragraphs. Specifically, we removed paragraphs with less than 5 words or consisting of short lines. Moreover, we only retained paragraphs with at least one temporal expression, as these would allow subsequent date or age extraction. As a result, we obtained a positive class prevalence of 7%.

Paragraphs were pre-processed by tokenising, lowercasing and removing punctuation. Two different text representations were compared: term frequency-inverse document frequency (TF-IDF) counts and word embeddings. For TF-IDF counts, we selected a minimum document frequency of 5 and a maximum of 5,000 features. For embeddings features, we used vectors pre-trained on CRIS discharge summaries^22^ and represented each paragraph by averaging all word vectors. As additional features, we also considered: paragraph length, number of symptom keywords (from list B), number of temporal expressions, earliest date and longest duration (as extracted by SUTimeMentalHealth).

In the cross-validation experiments, we considered three supervised classifiers: LR, RF, and SVM. To account for the class imbalance problem, we experimented with two different approaches: applying random subsampling on the majority class, and employing balanced class weights on all training data. In our experiments, using random subsampling resulted in the best recall results. Of note, we also performed experiments with a neural network approach based on long short-term memory (LSTM). However, probably due to the small size of our dataset, we were not able to obtain useful results and therefore discarded this model. In Fig. 5, relevant paragraphs that are correctly identified by the NLP classifier are marked in green (true positives), while non-relevant paragraphs are marked in yellow (false positives).

To combine extracted paragraphs on a patient level, we developed an heuristic approach, selecting one date or age for each paragraph and producing a ranked list of dates. The date selection was performed among the temporal expressions identified by SUTimeMentalHealth (an average of 3.5 expressions per paragraph): for each paragraph, we retained the earliest date and age (with year granularity). All temporal references where then ranked according to the classifier probability (for the associated paragraph), thus producing the final patient-specific list. In Fig. 5, the ranking is shown alongside each extracted paragraph (1: highest probability).

### NLP approach evaluation

For onset mention spans, IAA was evaluated on paragraphs: we considered true positives as paragraphs where both annotators marked an onset mention. For the Time_information and Type attributes, agreement was computed on overlapping mentions. Performance was measured with precision, recall, and F1 score.

In terms of NLP development, our end goal was to extract onset dates on a patient level. For the paragraph classification step, we used recall as the main performance metric. Patient-level aggregation was evaluated on adjudicated patients, using the annotated onset dates as the reference standard. If the top-N dates or ages identified by the NLP system matched at least one reference date, we considered this to be a match (N = [1, 3, 5]). All other cases were regarded as errors.

### Large-scale application

Our approach was validated at scale using CRIS data for the period 01/01/2007–31/12/2019. We extracted all first referral documents for patients with a diagnosis of schizophrenia, and applied the described filtering and NLP steps. Predicted onset dates were filtered by removing dates prior to 1900, the year of the Mental Health Act (1983), the current age of the patient, sentences related to relatives, and dates that happened after the first referral date. This led to 2483 patients with at least one predicted onset date, with 75% of them having a maximum of 3 dates. We considered 3 possible onset dates per patient, and calculated corresponding DUP values by subtracting these dates from the first referral date.

To verify whether our NLP approach for onset extraction was resulting in reasonable predictions, we compared minimum DUP values for patients seen by FEP teams vs. other teams. For each patient, we looked at teams where the acceptance date was within three months of the first referral date. To identify FEP teams, we used a list of team names provided by one practising psychiatrist.

### Ethical approval

The de-identified CRIS database has received ethical approval for secondary analysis: Oxford REC C, reference 18/SC/0372. CRIS data is made available to approved researchers working on approved projects under a rigorous governance model. Projects are approved by the CRIS Oversight Committee, a body setup by and reporting to the SLaM Caldicott Guardian. Researchers are approved by application to SLaM NHS Trust.
